# Systemic therapy targeting psoriatic inflammation is associated with decreased incidence of dementia: An observational retrospective cohort study

**DOI:** 10.1016/j.jdin.2025.06.006

**Published:** 2025-07-16

**Authors:** Madison P. Olexson, Ana Ormaza Vera, Chunghwan Ro, Clinton W. Enos

**Affiliations:** a Department of Dermatology, Eastern Virginia Medical School, Macon & Joan Brock Virginia Health Sciences at Old Dominion University, Norfolk, Virginia, USA; b St. George's University School of Medicine, St. George's, Grenada

**Keywords:** Alzheimer disease, biologics, dementia, inflammatory, psoriasis, vascular dementia

*To the Editor:* Psoriasis, an immune-mediated chronic inflammatory skin disorder, has been implicated in cognitive impairment and dementia.^1^, ^2^, ^3^ Emerging evidence suggests that systemic inflammation contributes to neuroinflammation and resultant cytokine-driven neurodegeneration, although findings remain inconclusive.^1^, ^2^, ^3^ While systemic therapies such as biologics and conventional disease-modifying antirheumatic drugs are routinely used in psoriasis management, their role in modulating dementia in this population remains underexplored, with research yielding mixed results.^1^, ^2^, ^3^ We conducted a large-scale, retrospective cohort study to investigate the relationship between psoriasis and dementia and whether systemic treatments for psoriasis impact this association.

We used deidentified data from the TriNetX Research Network between 2005 and 2025. Participants were identified using the International Classification of Diseases 10th revision Clinical Modification (ICD-10-CM) codes (Supplementary Table I, available via Mendeley at https://data.mendeley.com/datasets/667w56dkb7/1). Individuals aged 65-95 years without a prior history of dementia were included. A control cohort of individuals without psoriasis was compared with 2 experimental cohorts: (1) treated psoriasis patients (ie, receiving systemic therapies) (Supplementary Tables II-IX, available via Mendeley at https://data.mendeley.com/datasets/667w56dkb7/1) and (2) untreated psoriasis patients (ie, managed without systemic therapies). Cohorts were 1:1 propensity score-matched by demographics, body mass index, and risk factors for dementia. Primary outcomes were the incidence of Alzheimer disease, vascular, and nonvascular dementia assessed over a period of up to 20 years. Odds ratios (ORs) with 95% CIs were calculated, with a stratified analysis based on systemic treatment.

We identified 4,378,994 patients without psoriasis, 45,713 untreated psoriasis patients, and 14,664 treated psoriasis patients (Supplementary Fig 1, available via Mendeley at https://data.mendeley.com/datasets/667w56dkb7/1). After meeting the index event inclusion criteria and 1:1 propensity score-matching, cohorts were matched to their respective nonpsoriasis controls (Supplementary Tables III-VIII). Over a 20-year follow-up, untreated psoriasis patients were >33% more likely to develop Alzheimer disease (668 vs 474; OR = 1.42 [1.26-1.60]), vascular (415 vs 313; OR = 1.33 [1.15-1.54]), and nonvascular dementia (1,985 vs 1,498; OR = 1.35 [1.26-1.44]) compared with nonpsoriasis patients (Table I). Conversely, treated psoriasis patients were 38% less likely to develop Alzheimer disease (117 vs 189; OR = 0.62 [0.49-0.78]), 32% less likely to develop vascular dementia (85 vs 124; OR = 0.68 [0.52-0.90]), and 18% less likely to develop nonvascular dementia (454 vs 554; OR = 0.82 [0.72-0.93]) compared with nonpsoriasis controls (Table I). A subgroup analysis demonstrated that psoriasis patients receiving any systemic treatment (eg, biologics and nonbiologics) have a significantly reduced likelihood of developing dementia compared to untreated psoriasis patients (Table II).

**Table I:** Dementia outcomes among psoriasis patients aged 65 to 95 years with and without systemic therapy vs nonpsoriasis controls over a 20-year follow-up period

Outcome	Unadjusted incidence		Adjusted incidence		Odds ratio (95% CI)	
	Untreated∗ psoriasis (*n* = 34,790)	Nonpsoriasis (*n* = 3,997,089)	Untreated psoriasis (*n* = 34,790)	Nonpsoriasis (*n* = 34,790)	Unadjusted	Adjusted
Alzheimer disease, *n* (%)	668 (1.9)	75,610 (1.6)	668 (1.9)	474 (1.4)	1.17 (1.08-1.26)	1.42 (1.26-1.60)
Vascular dementia, *n* (%)	415 (1.2)	41,185 (1.0)	415 (1.2)	313 (0.9)	1.16 (1.05-1.28)	1.33 (1.15-1.54)
Nonvascular dementia‡, *n* (%)	1985 (5.7)	201,776 (5.0)	1985 (5.7)	1498 (4.3)	1.14 (1.09-1.19)	1.35 (1.26-1.44)


	Treated† psoriasis (*n* = 13,656)	Nonpsoriasis (*n* = 3,997,089)	Treated psoriasis (*n* = 13,656)	Nonpsoriasis (*n* = 13,656)		
Alzheimer disease, *n* (%)	117 (0.86)§	65,873 (1.6)	117 (0.86)§	189 (1.4)	0.52 (0.43-0.62)	0.62 (0.49-0.78)
Vascular dementia, *n* (%)	85 (0.62)§	41,185 (1.0)	85 (0.62)§	124 (0.91)	0.60 (0.49-0.75)	0.68 (0.52-0.90)
Nonvascular dementia, *n* (%)	454 (3.3)§	201,776 (5.0)	454 (3.3)§	554 (4.1)	0.65 (0.59-0.72)	0.82 (0.72-0.93)

∗ Those not receiving systemic therapies: methotrexate, apremilast, acitretin, adalimumab, etanercept, infliximab, certolizumab pegol, golimumab, ustekinumab, guselkumab, tildrakizumab, risankizumab, secukinumab, ixekizumab, and brodalumab.

† Those receiving any systemic therapy: methotrexate, apremilast, acitretin, adalimumab, etanercept, infliximab, certolizumab pegol, golimumab, ustekinumab, guselkumab, tildrakizumab, risankizumab, secukinumab, ixekizumab, and brodalumab.

‡ International Classification of Diseases (ICD) codes including Alzheimer disease (ICD-10-G30), frontotemporal dementia (ICD-10-G31.0), dementia in other diseases classified elsewhere (ICD-10-F02), and unspecified dementia (ICD-10-F03).

§ Among treated psoriasis patients, 26 with Alzheimer disease, 17 with vascular dementia, and 90 with nonvascular dementia were excluded from the results because they had the outcome prior to the 20-year time window.

**Table II:** Dementia outcomes among psoriasis patients aged 65-95 years on any systemic therapy, biologic therapy, or nonbiologic therapy vs psoriasis patients not receiving treatment over a 20-year follow-up period

Outcome	Unadjusted incidence		Adjusted incidence		Odds ratio (95% CI)	
	Treated∗ psoriasis (*n* = 13,656)	Untreated† psoriasis (*n* = 34,790)	Treated psoriasis (*n* = 13,619)	Untreated psoriasis (*n* = 13,619)	Unadjusted	Adjusted
Alzheimer disease, *n* (%)	117 (0.86)¶	668 (1.9)	116 (0.85)¶	235 (1.7)	0.44 (0.36-0.54)	0.49 (0.39-0.61)
Vascular dementia, *n* (%)	85 (0.62)¶	415 (1.2)	85 (0.63)¶	150 (1.1)	0.52 (0.41-0.66)	0.57 (0.43-0.74)
Nonvascular dementia‡, *n* (%)	454 (3.3)¶	1985 (5.7)	453 (3.3)¶	726 (5.3)	0.57 (0.52-0.64)	0.62 (0.55-0.69)


	Biologics§ (*n* = 3857)	Untreated psoriasis (*n* = 34,790)	Biologics (*n* = 3833)	Untreated psoriasis (*n* = 3833)		
Alzheimer disease, *n* (%)	26 (0.68)#	668 (1.9)	25 (0.65)#	65 (1.7)	0.35 (0.23-0.51)	0.38 (0.24-0.61)
Vascular dementia, *n* (%)	20 (0.52)#	415 (1.2)	20 (0.52)#	45 (1.2)	0.43 (0.28-0.68)	0.44 (0.26-0.75)
Nonvascular dementia, *n* (%)	101 (2.6)#	1985 (5.7)	99 (2.6)#	201 (5.2)	0.45 (0.37-0.55)	0.48 (0.38-0.62)


	Nonbiologics‖ (*n* = 6175)	Untreated psoriasis (*n* = 34,790)	Nonbiologics (*n* = 6171)	Untreated psoriasis (*n* = 6171)		
Alzheimer disease, *n* (%)	60 (0.98)∗∗	668 (1.9)	60 (0.98)∗∗	128 (2.1)	0.50 (0.39-0.66)	0.47 (0.34-0.63)
Vascular dementia, *n* (%)	44 (0.71)∗∗	415 (1.2)	44 (0.71)∗∗	87 (1.4)	0.60 (0.44-0.81)	0.50 (0.35-0.72)
Nonvascular dementia, *n* (%)	234 (3.8)∗∗	1985 (5.7)	234 (3.8)∗∗	389 (6.3)	0.66 (0.57-0.75)	0.59 (0.50-0.70)

∗ Receiving any systemic therapy: methotrexate, apremilast, acitretin, adalimumab, etanercept, infliximab, certolizumab pegol, golimumab, ustekinumab, guselkumab, tildrakizumab, risankizumab, secukinumab, ixekizumab, and brodalumab.

† Not receiving systemic therapy: methotrexate, apremilast, acitretin, adalimumab, etanercept, infliximab, certolizumab pegol, golimumab, ustekinumab, guselkumab, tildrakizumab, risankizumab, secukinumab, ixekizumab, and brodalumab.

‡ International Classification of Diseases (ICD) codes including Alzheimer disease (ICD-10-G30), frontotemporal dementia (ICD-10-G31.0), dementia in other diseases classified elsewhere (ICD-10-F02), and unspecified dementia (ICD-10-F03).

§ Adalimumab, etanercept, infliximab, certolizumab pegol, golimumab, ustekinumab, guselkumab, tildrakizumab, risankizumab, secukinumab, ixekizumab, and brodalumab.

‖ Methotrexate, apremilast, and acitretin.

¶ Among treated psoriasis patients, 26 with Alzheimer disease, 17 with vascular dementia, and 90 with nonvascular dementia were excluded from the results because they had the outcome prior to the 20-year time window.

# Among psoriasis patients on a biologic, 10 with Alzheimer disease, 10 with vascular dementia, and 26 with nonvascular dementia were excluded from the results because they had the outcome prior to the 20-year time window.

∗∗ Among psoriasis patients on a nonbiologic, 19 with Alzheimer disease, 10 with vascular dementia, and 51 with nonvascular dementia were excluded from the results because they had the outcome prior to the 20-year time window.

Results from this study suggest a potential association between systemic psoriasis treatments and a reduced likelihood of dementia. Interleukin-17 has been implicated in blood-brain barrier disruption and amyloid beta production,^4^ whereas tumor necrosis factor-α has been associated with impaired amyloid clearance and accelerated cognitive decline,^2^^,^^5^ highlighting the relevance of targeting these pathways. Although these findings raise important questions about the potential neurocognitive benefits of immunomodulatory treatments, they should be interpreted cautiously given the inherent limitations of observational database research. This study is subject to potential selection bias and residual confounding, including unmeasured factors such as socioeconomic status, health care literacy, and disease severity, which may influence the likelihood of receiving systemic treatment and dementia outcomes. Additionally, since systemic therapy was treated as an ever/never binary exposure, immortal-time and healthy-user biases are limitations as well.

In conclusion, untreated psoriasis appears to associate with an increased likelihood of dementia compared with controls, whereas systemic anti-inflammatory therapy is associated with a decreased odds of dementia outcomes in psoriasis patients. These results underscore the need for prospective, controlled studies to evaluate potential neurocognitive effects of systemic psoriasis treatments.

## Conflicts of interest

Dr Enos has previously served as a consultant on an advisory board for UCB and Amgen. He is an investigator for Amgen and Castle Biosciences. He receives grant funding from La Roche-Posay and has previously received research funding from the ASA/Arcutis Biotherapeutics. Olexson, Dr Ormaza Vera, and Ro have no conflicts of interest to declare.
